# Hypolipidemic and Antioxidant Effects of Dandelion (*Taraxacum officinale*) Root and Leaf on Cholesterol-Fed Rabbits

**DOI:** 10.3390/ijms11010067

**Published:** 2010-01-06

**Authors:** Ung-Kyu Choi, Ok-Hwan Lee, Joo Hyuk Yim, Chang-Won Cho, Young Kyung Rhee, Seong-Il Lim, Young-Chan Kim

**Affiliations:** 1 Pohang Center for Evaluation of Biomaterials, Pohang 790-834, Korea; E-Mail: cuk8272@hanmail.net (U.-K.C.); 2 Department of Biomedical Science, CHA University, Seongnam, Kyonggi, 463-836, Korea; 3 Korea Food Research Institute, Seongnam, Kyonggi, 463-746, Korea; E-Mails: jlunar@naver.com (J.H.Y.); cwcho@kfri.re.kr (C.-W.C.); ykrhee@kfri.re.kr (Y.K.R.); silim@kfri.re.kr (S.-I.L.)

**Keywords:** dandelion, Taraxacum officinale, atherosclerosis, antioxidant activity, hypolipidemic effects

## Abstract

Dandelion (*Taraxacum officinale*), an oriental herbal medicine, has been shown to favorably affect choleretic, antirheumatic and diuretin properties. Recent reports have indicated that excessive oxidative stress contributes to the development of atherosclerosis-linked metabolic syndrome. The objective of this current study was to investigate the possible hypolipidemic and antioxidative effects of dandelion root and leaf in rabbits fed with a high-cholesterol diet. A group of twenty eight male rabbits was divided into four subgroups; a normal diet group, a high-cholesterol diet group, a high-cholesterol diet with 1% (w/w) dandelion leaf group, and a high-cholesterol diet with 1% (w/w) dandelion root group. After the treatment period, the plasma antioxidant enzymes and lipid profiles were determined. Our results show that treatment with dandelion root and leaf positively changed plasma antioxidant enzyme activities and lipid profiles in cholesterol-fed rabbits, and thus may have potential hypolipidemic and antioxidant effects. Dandelion root and leaf could protect against oxidative stress linked atherosclerosis and decrease the atherogenic index.

## 1. Introduction

Atherosclerotic disease causes over 19 million deaths annually, yet our understanding of the fundamental aspects of the genesis of the disease remains incomplete [1,2]. Several studies have shown that an increased dietary intake of cholesterol results in hypercholesterolemia, which is known to eventually generate atherosclerosis and enhance the risk of coronary heart disease (CHD), fatty liver disease and cancer associated with hydroxyl radical formation [3,4]. The etiology of atherosclerosis appears to be a multi-factorial series of events, but the oxidation of lipoprotein is believed to be a primary event in the pathogenesis of atherosclerosis [5]. Thus, recent interest has been focused on strategies to enhance the removal of reactive oxygen species (ROS) by using antioxidants to enhance endogenous antioxidant responses.

Natural antioxidants protect dietary lipids from oxidation, but may also provide health benefits associated with preventing damage due to biological degeneration. Antioxidative substances are believed to suppress the onset and development of atherosclerosis and the compound such as probucol has been shown to reduce the progression of atherosclerosis lesions in Watanabe heritable hyperlipidemic rabbits (WHHR) *in vivo*. In addition, flavonoids and phenolic compounds have also been proved to have antioxidative effects. Thus, substances with both antioxidative and hypocholesterolemic properties are expected to be effective in preventing the formation and/or progression of atherosclerosis [6].

*Taraxacum officinale*, known as dandelion, has been used in folk medicine in the treatment of hepatic disorders, inflammation and several women’s diseases such as breast and uterus cancers. In Traditional Chinese medicine, it is also acclaimed as a nontoxic herb with exceptional values for its choleretic, diuretic, anti-rheumatic and anti-inflammatory properties. Several flavonoids including caffeic acid, chlorogenic acid, luteolin, and luteolin 7-glucoside have been isolated from the dandelion [7]. However, relatively less has been studied about the preventive effect of dandelion root and leaf on atherosclerosis. This present study was therefore to evaluate the potentials of orally administered dandelion root and leaf on the development of atherosclerosis by the evaluation of antioxidant enzyme response and lipid profiles in high-cholesterol-fed rabbits.

## 2. Results and Discussion

### 2.1. Effects on weight gain and liver weight

As shown in Table 1, there was no statistically significant difference in weight gain between the treatment groups and normal group. Compared to the normal group, the liver weight was significantly increased in control group and dandelion leaf and root group. However, neither dandelion leaf nor root significantly affected the liver weight gain, compared with control group.

### 2.2. Effect on plasma enzyme and lipid profiles

The changes of plasma AST and ALT activities and lipid profile of rabbits that consumed different diets are shown in Tables 2 and 3. The plasma AST concentrations was slightly decreased in the dandelion leaf fed group compared to the control group. The ALT activity was significantly decreased in the dandelion root fed group. Both dandelion leaf and root diets have increased the level of CK in rabbits consumed high-cholesterol diet. As shown in Table 3, dandelion leaf supplementation resulted in significant increase in the levels of HDL cholesterol. The diet with dandelion leaf also lowered the both levels of triglyceride and LDL cholesterol significantly. Triglyceride level was significantly lower in the dandelion root group than in the control group.

### 2.3. Effects on the hepatic antioxidant enzymes activities and liver lipid peroxidation

The effects of dandelion supplementation on hepatic antioxidant enzyme activities are shown in Table 4. GSH activities were significantly increased in both dandelion leaf and root groups, compared to the control group. Supplementation with dandelion root significantly decreased the GST activities compared to the control group (*p* < 0.05). Non significant reduction in GST activity was shown in dandelion leaf group. Diet either with dandelion leaf or root increased GPx and SOD activities in hepatic tissues, respectively. However, there are significantly lower the CAT activities in dandelion leaf or root than control. In the respect of lipid peroxidation in liver, treatment with dandelion root resulted in significant reduction in the level of TBARS. Supplementation with dandelion leaf showed no significant reduction in TBARS level.

### 2.4. Morphological changes of rabbit aorta

**Figure 1.**
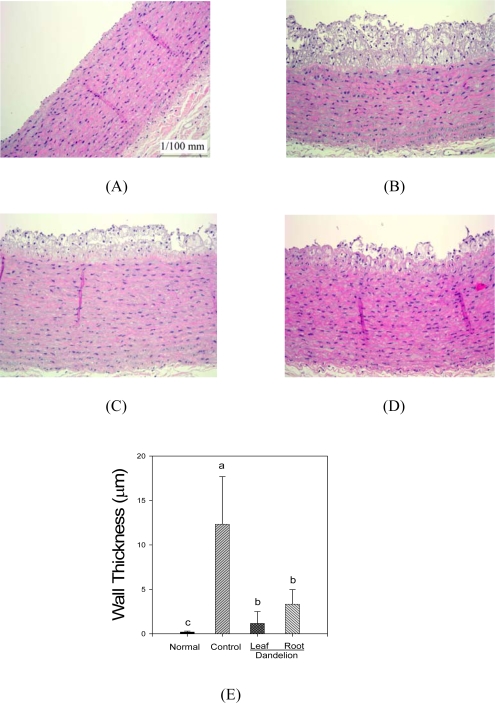
The aortic sections of rabbits fed different diets. (A) normal diet, (B) high cholesterol diet, (C) high cholesterol + 1% (w/w) dandelion leaf diet, (D) high cholesterol + 1% (w/w) dandelion root diet, (E) The graph shows data of means ± standard deviation from seven animals per group.^a–c^ Means in the same column not sharing a common letter are significantly different (*P* < 0.05) by Duncan’s multiple test.

Representative aortic sections stained with hematoxylin-eosin and obtained from each group are shown in Figure 1. As shown in group (A) in the figure, the aortic walls in the control rabbits were smooth and intact. By feeding high cholesterol diet was developed a thick layer of lipid deposition within intima of aorta which is typical for atherosclerosis. The formations of atherosclerotic lesions were notably decreased by the supplementation with either dandelion leaf or root.

Treatment with the dandelion leaf and root resulted in positive changes of plasma enzyme activities and lipid levels, except CK and phospholipids. Not only the atherogenic index, which is recognized as a better arteriosclerosis marker than each of the lipid parameters alone, but they also prevented oxidative damage, an important etiologic factor in atherosclerosis. The relationship between ALT, as a possible marker of liver disorders including steatosis, and CHD risk factors, including the elements of the metabolic syndrome, has been shown in previous studies [7–10]. AST activity was significantly decreased in the dandelion leaf group compared to the control group (*p* > 0.05), ALT activity was significantly decreased in the dandelion root group.

We have recently reported [11] the total phenol contents of the dandelion leaf and root were 7.9 ± 0.4% and 9.4 ± 0.3%, respectively. We also reported in this study that the cathechol, caffeic acid, ferulic acid, m-coumaric acid, *p*-coumaric acid, vanillic acid and syringic acid were found to be phenolic compounds in the dandelion leaf and root, which is consistent with other reports [7].

In the human body, high levels of triglycerides in the bloodstream have been linked to atherosclerosis, and, by extension, the risk of heart disease and stroke. TG level was significantly decreased in the experimental groups (dandelion leaf and root) compared to the control group (*P* > 0.05). High cholesterol levels are strong indicators of those individuals that are prone to coronary heart disease. Elevated total cholesterol is a risk factor for coronary heart disease. The build-up of plaque in the artery may lead to narrowing (high blood pressure) or complete blockage (heart attack) of the vessel [12]. It is widely accepted that reduction in plasma HDL is a risk factor for developing atherosclerosis. HDL facilitates the translocation of cholesterol from the peripheral tissue, such as arterial walls to liver for catabolism. The increase in HDL-C may slow down the atherosclerotic process [13]. Atherosclerotic index (AI), defined as the ratio of LDL-C and HDL-C, is believed to be an important risk factor of atherosclerosis [14]. Our results showed that the dandelion leaf supplemented-diet increased the concentrations of serum HDL-C when is compared with the cholesterol-rich diet, and the concentrations of serum LDL-C were decreased.

Oxidative stress is one of the causative factors that link hypercholesterolemia with atherogenesis [15]. A cholesterol-rich diet brings about remarkable modifications in antioxidant defense mechanisms. In addition to, recently report shown that hypercholesterolemia diminishes the antioxidant defense system and decreases the activities of SOD and CAT, elevating the lipid peroxide content [16]. The present results are in agreement with this theory. The oxidative parameters of only the cholesterol-fed rabbit (control group) were good results compared with these of the cholesterol-non fed rabbits (normal group). GST is the important detoxification enzyme involved in catalyzing the conjugation of a wide variety of electrophilic substrates to reduced glutathione and thus protects the cell from chemically induced damages in hepatic and extra hepatic tissues [17]. GST contents increases when one is sick. GST of dandelion root group decreases activities compared with control group. GPx is more important than catalase for detoxification of hydrogen peroxide in brain, because the brain contains small amounts of catalase and GPx can also interact directly with lipid peroxides [18,19].The result of GPx and CAT analysis significantly increased in the experimental groups (dandelion leaf and root) compared to the control group (*P* > 0.05). Especially, the dandelion root group was significantly high antioxidant enzyme activity better than the dandelion leaf (*P* > 0.05).

MDA, the product of lipid peroxidation, is an index of the level of oxygen free radicals. A decrease in lipid peroxidation leads to a reduction of atherosclerosis caused by hypercholesterolemia [20]. The content of MDA in rabbits fed a cholesterol-rich diet and a dandelion leaf diet were increased compared to rabbits fed standard laboratory diet, suggesting that hypercholesterolemia could enhance the process of lipid peroxidation. The dandelion leaf prevented a cholesterol-rich diet which induced elevation of MDA and resulted in a significantly decreased content of MDA in liver. The obtained data suggested that dandelion root might be capable of lowering or slowing down oxidative-related lipid peroxidation.

The thickening of the vascular wall and infiltration of macrophages and lymphocytes are hallmarks of atherosclerosis [21]. In the result of histopathological examination, the formation of atheromatous plaque aortic media notably were decreased in the experimental groups (dandelion leaf and root) compared to the control group. In the case of serum enzyme activities and lipid levels the effect of the dandelion leaf is better, while in the case of the hepatic antioxidant activities the effect of the dandelion root is better. Hypercholesterolemia with high cholesterol diet was associated with development of atherosclerosis. These findings are consistent with the previous [22,23]. Dandelion reduced the development of atherosclerosis. Hypercholesterolemic atherosclerosis was associated with increases of ROS suggesting increased levels of oxygen radicals. Increased levels of oxygen radicals are known to produce endothelial cell injury, which represents a critical initiating event in the development of atherosclerosis [24]. Dandelion may have prevented the oxygen radical-induced endothelial cell injury through its antioxidant activity. The reduction in the extent of atherosclerosis could also be related to its lipid lowering activity.

## 3. Experimental Section

### 3.1. Plant material

Dandelion leaves and roots obtained from Mindule Food Inc. (Uiryeong, Gyeongbuk, Korea). A voucher specimen of the plant materials (voucher No. DAN-1) was deposited in the Region Food Industry Research Group (Korea Food Research Institute, Gyeonggi, Korea). Fresh dandelion leaf and root were washed with distilled water, and dried with air. The dried dandelion leaf and root were ground to a fine powder using a grinder (IKA M 20, IKA, Staufen, Germany).

### 3.2. Animals and diets

Twenty eight male New Zealand white rabbits (16 weeks, 1.8~2.2 kg) were purchased from Central Lab. Animal Inc. (Seoul, Korea). The animals were individually housed in stainless-steel cages in an air-conditioned room (19 ± 1 °C, 55 ± 5% humidity) with 12 h light/dark cycle. They were randomly divided into four groups: normal, control, dandelion leaf and root-supplemented groups (Table 5). Rabbits fed a normal diet were used as the normal control. All rabbits (except normal control) were fed a hypercholesterolemic diet containing rabbit chow (Purina, Seoul, Korea) enriched with 1% (w/w) cholesterol (Sigma, St. Louis, MO, USA). All rabbits were initially fed a normal diet for one week and then randomly divided into a hypercholesterolemic control group (n = 7, 1% high cholesterol diet) and two treatment groups (n = 7, 1% high cholesterol diet along with 1% dandelion leaf and root, respectively). Food intake for each rabbit was standardized to 250 g/day throughout the experiment of 4 weeks. This experiment was conducted in facilities approved by the Association for Assessment and Accreditation of Laboratory Animal Care (AAALAC) International. All procedures were approved by our Institutional Animal Care and Use Committee (IACUC).

### 3.3. Experimental procedure

The body weight gain was measured once a week and blood samples were collected from retro-orbital venous plexus puncture. The plasma triglycerides (TG) total cholesterol (TC), high density lipoprotein (HDL)- and low density lipoprotein (LDL)- cholesterol, aspartate aminotransferase (AST), alanine aminotransferase (ALT), creatine kinase and phospholipids were measured by Green Cross Reference (Gyeonggi, Korea). At the end of the experimental period, the rabbits were anesthetized with ether after a 16 h fast. The abdominal aorta, including the aortic arch was rinsed with physiological saline and stored in 10% buffered neutral formalin. Blood was drawn from retro-orbital venous plexus puncture. The liver was removed and rinsed with physiological saline, weighed (Ohaus, NJ, USA) and kept refrigerated until analysis. All the samples were stored at −70 °C until analyzed.

### 3.4. Preparation of liver homogenate

The liver was homogenized (Daihan, Seoul, Korea) in ice cold 0.1M PBS (pH 7.4) solution. The homogenates were centrifuged (Hanil, Seoul, Korea) at 10,000 × g for 1h at 4 °C to remove cell debris, nuclei, and mitochondria. Resulting supernatants were served for malondialdehyde (MDA) measurements. The protein content of the liver tissue was measured by Bradford protein assay kit (Bio Rad, Hercules, CA, USA).

### 3.5. GSH (Glutathione) activity

The content of GSH was measured by the method of Hissin and Half [25]. Phosphate-EDTA buffer (pH 8.0, 4.5 mL) was added to 0.5 mL of supernatant. The final assay mixture (2.0 mL) contained 100 μL of the diluted tissue supernatant, 1.8 mL of phosphate-EDTA buffer, and 100 μL OPT (*o*-phthalaldehyde) solution. After mixing and incubation at room temperature for 15 min, the solution was transferred to a quartz cuvette. Fluorescence at 420 nm was determined with the activation at 350 nm (Varian, Walnut Creek, CA, USA).

### 3.6. Glutathione-S-Transferase (GST) activity

GST activity was measured by the modified method of Habig *et al.* [26]. The mixture contained 500 μL of 0.2 M pH 6.5 phosphate buffer, 100 μL of 10 mM GSH, 200 μL of distilled water. The mixture added to 100 μL of sample and 100 μL of 10 mM 1-chloro-2,4-dinitrobenzene. After reaction 3 min, the absorbance was immediately read at 340 nm on a spectrophotomer (TS Science, Seoul, Korea).

### 3.7. Glutathione peroxidase (GPx) activity

GPx activity was determined by the method of Wendel [27] with modifications. The mixture contained 0.6 mL of 0.25 M pH 7.0 phosphate buffer, 0.3 mL of 10 mM GSH, 0.3 mL of 10 mM EDTA, 0.3 mL of 10 mM sodium azide, 0.3 mL of 2 mM NADPH and 20 μL glutathione reductase. The mixture was added to 0.9 mL of the sample and incubated (Vision, Gyeonggi Do, Korea) at 30 °C for 5 min. The reaction was initiated by the addition of 0.3 mL of 2.5 mM hydrogen peroxide (Sigma). The absorbance was immediately read at 340 nm.

### 3.8. Catalase (CAT) activity

The activity of CAT was assayed according to the method of Carrillo *et al.* [28] with minor modifications. The mixture 0.05 M pH 7.4 sodium phosphate buffer, 1 μM H_2_O_2_ and the sample was made up a final volume of 3 mL, and the decrease in absorbency was measured at 240 nm in 1 min. One unit of CAT activity was defined as the amount of enzyme required to decompose 1 μM of H_2_O_2_ in 1 min.

### 3.9. Superoxide dismutase (SOD) activity

The activity of SOD was measured according to the modified method of Carrillo *et al.* [28]. The enzyme was detected based on its ability to inhibit the superoxide-mediated reduction of cytochrome c by xanthine oxidase and xanthine. One hundred μL of the sample was added to 2 mL of 0.05 M pH 7.4 phosphate buffer, which contained 100 μM EDTA, 100 μM xanthine, 40 μM cytochrome c and 0.01 unit xanthine oxidase. The reaction was started by adding xanthine oxidase and then conducted at 30 °C for 3 min. The absorbance was measured at 550 nm. One enzyme unit was defined as the amount of enzyme required to inhibit the cytochrome c reduction by 50%.

### 3.10. Lipid peroxidation in liver

Malondialdehyde (MDA) levels were determined by monitoring thiobarbituric acid reactive substances (TBARS) according to the method of Ohkawa *et al.* [29] with minor modifications. The reaction mixture contained 0.2 mL of test sample, 0.2 mL of 8.1% sodium dodecyl sulfate (SDS), 1.5 mL of acetic acid and 1.5 mL of 0.5% of TBA. The mixture was heated in a water bath at 95 °C for 60 min. After cooling, 5 mL of *n*-butanol/pyridine (15:1, v/v) was added and the mixture shaken well. After centrifugation at 4,000 rpm for 10 min, the absorbance of the organic layer was measured at 532 nm.

### 3.11. Histopathological examination

The histopathological examination was performed by Chemon (Gyeonggi Do, Korea).

### 3.12. Statistical analysis

All data were expressed as mean ± SD. The data were assessed by ANOVA using SAS program (SAS institute, Cary, NC, USA) and the differences between the means were evaluated by Duncan’s multiple-range test. Statistical significance was considered at *p* < 0.05.

## 4. Conclusions

Based on the above results, these results suggest that diet-induced hypercholesterolemic atherosclerosis is associated with an increase in the oxidative stress and that dandelion reduced the extent of atherosclerosis by reducing oxidative stress and serum TC, TG, LDL-C and raising serum HDL-C. Dandelion is beneficial in preventing hypercholesterolemic atherosclerosis and reducing risk factors for coronary artery disease.

## Figures and Tables

**Table 1.** Effects of dandelion on body and liver weights of rabbits fed a high-cholesterol diet.

**Group ^(1)^**	**Body weight (kg/4 weeks)**	**Liver weight (g/4 weeks)**

Normal	2.451 ± 0.061 ^ns,(2)^	66 ± 5.35 ^b^
Control	2.467 ± 0.067	103.75 ± 6.19 ^a^
Dandelion Leaf	2.430 ± 0.198	105.25 ± 15.56 ^a^
Dandelion Root	2.368 ± 0.081	99.75 ± 12.45 ^a^

(1) Normal: normal diet, Control: high cholesterol diet, Dandelion leaf: high cholesterol + 1% dandelion leaf, Dandelion root; high cholesterol + 1% dandelion root.

(2) Means ± standard deviation (n = 7); means followed by different letters in the same row differ significantly (*P* < 0.05) (ns; not significant).

**Table 2.** Effects of dandelion supplementation on AST, ALT, CK concentration in serum in rabbits fed a high-cholesterol diet.

**Parameter (U/L)**	**Group/diet^(1)^**			
	**Normal**	**Control**	**Dandelion leaf**	**Dandelion root**
AST	29.40 ± 4.669 ^(2),b^	36.2 ± 2.588 ^a^	32.2 ± 2.950 ^a,b^	35 ± 3.317 ^a^
ALT	39.67 ± 7.394 ^c^	50.5 ± 5.541 ^a^	53.75 ± 2.754 ^a^	44.5 ± 2.380 ^b^
CK	534.75 ± 179.32 ^b^	802 ± 185.45 ^b^	1823.5 ± 813.66 ^a^	1775.3 ± 512.66 ^a^

(1) Normal: normal diet, Control: high cholesterol diet, Dandelion leaf: high cholesterol + 1% dandelion leaf, Dandelion root; high cholesterol + 1% dandelion root

(2) Means ± standard deviation (n = 7); means followed by different letters in the same row differ significantly (*P* < 0.05).

**Table 3.** Effects of dandelion supplementation on plasma lipid profiles in rabbits fed a high-cholesterol diet.

**Parameter (mg/dL)**	**Group/diet ^(1)^**			
	**Normal**	**Control**	**Leaf**	**Root**
Total cholesterol	57.25 ± 2.99 ^(2),c^	1267.3 ± 23.19 ^a,b^	1260.75 ± 29.23 ^b^	1304.5 ± 32.55 ^a^
HDL-cholesterol	25.25 ± 0.96 ^a^	18.5 ± 2.08 ^b^	24 ± 2.94 ^a^	18.75 ± 2.50 ^b^
LDL-cholesterol	19 ± 2.94 ^d^	496.75 ± 8.26 ^b^	441.25 ± 29.55 ^c^	637 ± 19.17 ^a^
Triglycerides	41.5 ± 4.80 ^d^	118.75 ± 4.43 ^a^	75.5 ± 3.70 ^c^	97.75 ± 9.46 ^b^
Phospholipids	85.75 ± 2.06 ^c^	419.5 ± 48.39 ^b^	498.75 ± 22.13 ^a,b^	573 ± 45.07 ^a^

(1) Normal: normal diet, Control: high cholesterol diet, Dandelion leaf: high cholesterol + 1% dandelion leaf, Dandelion root; high cholesterol + 1% dandelion root

(2) Means ± standard deviation (n = 7); means followed by different letters in the same row differ significantly (*P* < 0.05).

**Table 4.** Effect of dandelion supplementation on antioxidant enzymes in liver tissue of rabbits fed high-cholesterol diet.

**Parameters**	**Group/diet ^(1)^**			

	**Normal**	**Control**	**Dandelion leaf**	**Dandelion root**
GSH (mg/g liver)	5.145 ± 0.269 ^(2),a^	3.527 ± 0.085 ^b^	4.766 ± 0.223 ^a^	4.842 ± 0.458 ^a^
GST (unit/mg protein/min)	2.849 ± 0.370 ^c^	5.023 ± 0.380 ^a^	4.445 ± 0.293 ^a^	3.734 ± 0.190 ^b^
GPx (unit/mg protein/min)	97.88 ± 8.456 ^a^	54.54 ± 3.884 ^d^	74.75 ± 5.026 ^c^	86.51 ± 5.639 ^b^
CAT (unit/mg protein/min)	1.178 ± 0.329 ^d^	9.238 ± 0.074 ^a^	3.863 ± 0.240 ^c^	5.854 ± 0.179 ^b^
SOD (unit/mg protein/min)	0.5 ± 0.024 ^a^	0.294 ± 0.018 ^b^	0.494 ± 0.029 ^a^	0.47 ± 0.051 ^a^
TBARS (μM/mg protein)	0.602 ± 0.018 ^b^	0.699 ± 0.032 ^a^	0.679 ± 0.011 ^a^	0.608 ± 0.018 ^b^

(1) Normal: normal diet, Control: high cholesterol diet, Dandelion leaf: high cholesterol + 1% dandelion leaf, Dandelion root; high cholesterol + 1% dandelion root

(2) Means ± standard deviation (n = 7); means followed by different letters in the same row differ significantly (*P* < 0.05).

**Table 5.** Composition of experimental diets.

**Ingredient**	**Groups**			
	**Normal (%)**	**Control (%)**	**Dandelion leaf (%)**	**Dandelion root (%)**
Crude protein	15.0	15.0	15.0	15.0
Crude fat	2.0	2.0	2.0	2.0
Crude fiber	15.0	15.0	15.0	15.0
Crude ash	7.8	7.8	7.8	7.8
Ca	1.2	1.2	1.2	1.2
P	1.0	1.0	1.0	1.0
Nitrogen free extract	58.0	58.0	58.0	58.0

Total	100.0	100.0	100.0	100.0

Cholesterol	-	1.0	1.0	1.0
Dandelion leaf	-	-	1.0	-
Dandelion root	-	-	-	1.0
